# Supplementary material for: The Nuclear Pore Complex Facilitates Centriole-Nuclear Attachment in Spermatids
Source: bioRxiv. 2026 May 1:2026.04.28.721503. Preprint. [Version 1] doi: 10.64898/2026.04.28.721503 (PMC13142455; doi:10.64898/2026.04.28.721503)
Supplement: Supplement 1 [file NIHPP2026.04.28.721503v1-supplement-1.pdf]

was not certified by peer review) is the author/funder. This article is a US Government work. It is not subject to copyright under 17 USC 105
and is also made available for use under a CC0 license.

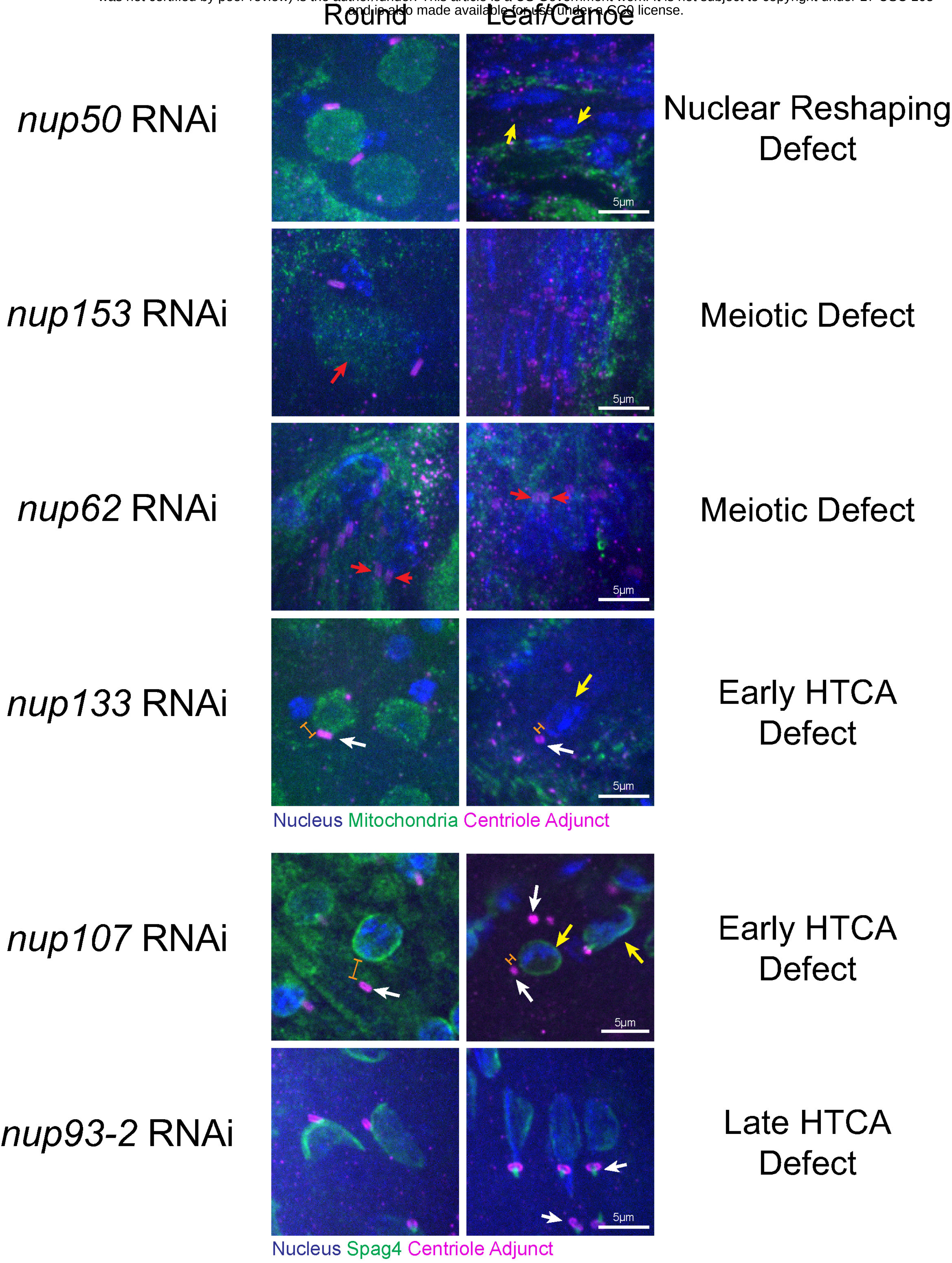

Buglak et al., Figure S1

bioRxiv preprint doi: https://doi.org/10.64898/2026.04.28.721503; this version posted May 1, 2026. The copyright holder for this preprint (which was not certified by peer review) is the author/funder. This article is a US Government work. It is not subject to copyright under 17 USC 105 and is also made available for use under a CC0 license.

Supplemental Figure 1: Nucleoporin RNAi screen revealed different phenotypes in spermiogenesis

Representative images showing the different phenotypes observed in the nucleoporin RNAi screen. All RNAi was driven by the testis-specific *Bam-Gal4* driver. Blue, nucleus (DAPI); magenta, centriole adjunct (Asl); green, mitochondria (ATP5A) or Spag4 (Spag4::6myc). Yellow arrows indicate nuclear shaping defects. Red arrows indicate meiotic defects with more than one centriole or nucleus in a single spermatid. White arrows indicate HTCA defects. Orange brackets indicate separation between nucleus and centriole. Scale bars 5um.

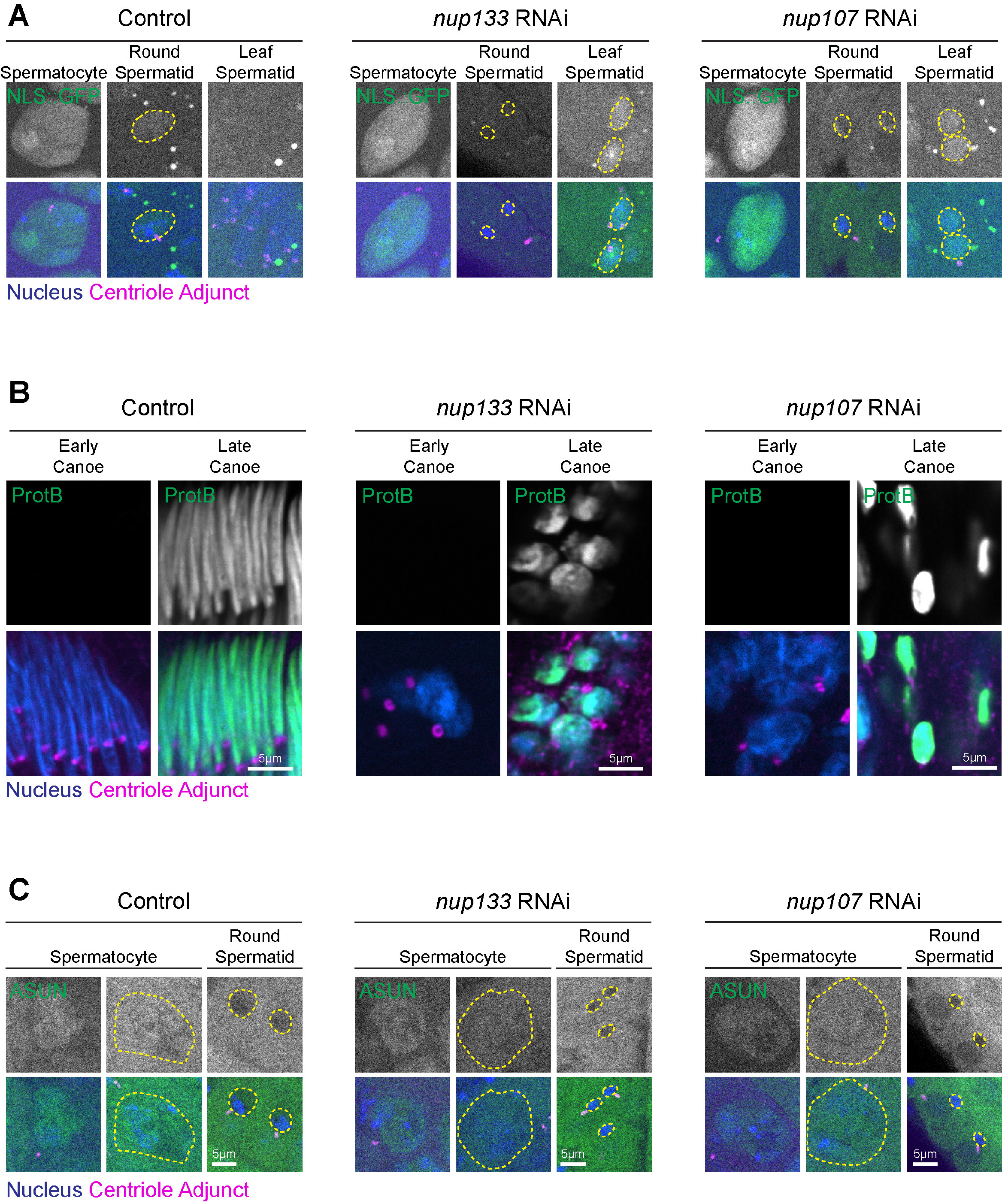

Buglak et al., Figure S2

bioRxiv preprint doi: https://doi.org/10.64898/2026.04.28.721503; this version posted May 1, 2026. The copyright holder for this preprint (which was not certified by peer review) is the author/funder. This article is a US Government work. It is not subject to copyright under 17 USC 105 and is also made available for use under a CC0 license.

Supplemental Figure 2: Nucleocytoplasmic transport still occurs following Nup depletion

(A) Representative images of Spermatocytes and Round and Canoe spermatids for the indicated genotypes. Blue, nucleus (DAPI); magenta, centriole adjunct (Asl); green, NLS::GFP. Dashed yellow line shows outline of nucleus. Scale bar 5um. **(B)** Representative images of Early Canoe and Late Canoe spermatids for the indicated genotypes. Blue, nucleus (DAPI); magenta, centriole adjunct (Asl); green, Protamine B (ProtB::GFP). Scale bar 5um. **(C)** Representative images of Spermatocytes and Round spermatids for the indicated genotypes. Blue, nucleus (DAPI); magenta, centriole adjunct (Asl); green, asunder (ASUN::GFP). Dashed yellow line shows outline of nucleus. Scale bar 5um.

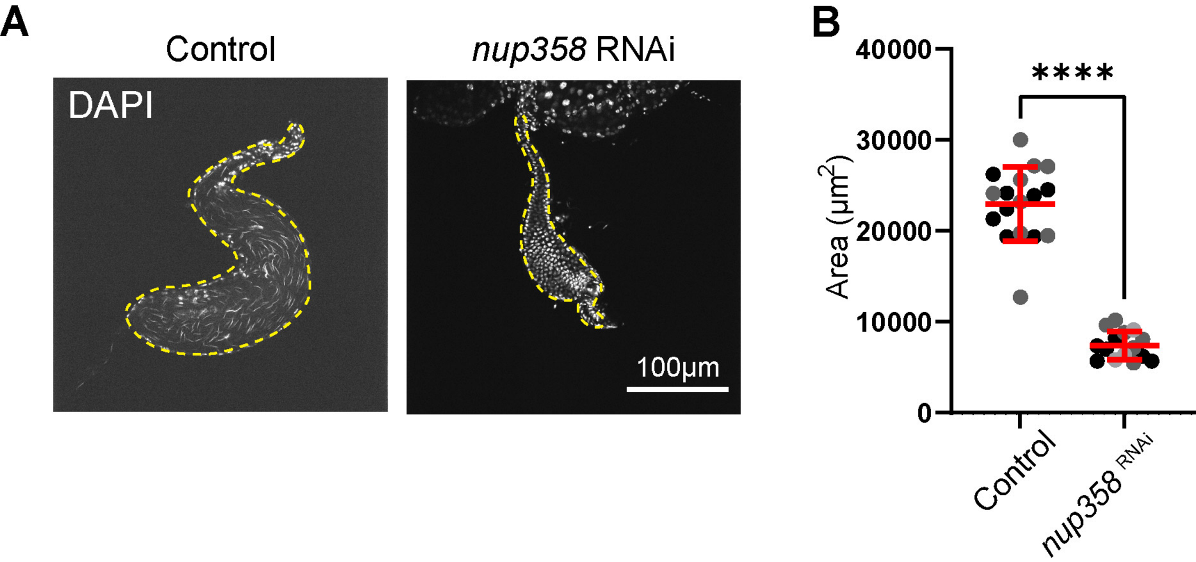

Buglak et al., Figure S3

bioRxiv preprint doi: https://doi.org/10.64898/2026.04.28.721503; this version posted May 1, 2026. The copyright holder for this preprint (which was not certified by peer review) is the author/funder. This article is a US Government work. It is not subject to copyright under 17 USC 105 and is also made available for use under a CC0 license.

Supplemental Figure 3: Nup358 is required for proper sperm production

(A) Representative images of seminal vesicles of the indicated RNAi knockdowns stained with DAPI (DNA). Yellow dashed outline shows seminal vesicles. Scale bar 100um. **(B)** Quantification of seminal vesicle area for controls (*Bam-Gal4/+,* n=17 seminal vesicles) and *nup358* RNAi (*nup358 RNAi/+; Bam-Gal4/+,* n=15 seminal vesicles); ****=$p \leq 0.0001$.

bioRxiv preprint doi: https://doi.org/10.64898/2026.04.28.721503; this version posted May 1, 2026. The copyright holder for this preprint (which was not certified by peer review) is the author/funder. This article is a US Government work. It is not subject to copyright under 17 USC 105 and is also made available for use under a CC0 license.

Table S1: Nucleoporin RNAi screen of HTCA phenotypes

Nup	Type	RNAi line	Bam-Gal4 driven phenotype
Nup50	Nuclear basket	#34580 (Bloomington)	Impaired nuclear shaping, possible HTCA defect
Nup153	Nuclear basket	#30504 (Bloomington)	Meiotic defect
Mgtor (tpr)	Nuclear basket	#32941 (Bloomington)	Normal
Ndc1	Transmembrane ring	#67275 (Bloomington)	Multiple centrioles; impaired nuclear shaping
Gp210	Transmembrane ring	#65219 (Bloomington)	Normal
Nup54	Central channel	#57426 (Bloomington)	Normal
Nup58	Central channel	#60110 (Bloomington)	Impaired nuclear shaping
Nup62	Central channel	#35695 (Bloomington)	Meiotic defect; multiple centrioles
Nup98-96	Central channel	#28562 (Bloomington)	Normal
Nup93-2	Inner ring	#51758 (Bloomington)	Late HTCA defect; impaired nuclear shaping
Nup35	Inner ring	#66002 (Bloomington)	Normal
Nup154 (Nup155)	Inner ring	#34710 (Bloomington)	Severe meiotic defect; HTCA defect
Nup205	Inner ring	#28610 (VDRC)	Meiotic defect/multiple centrioles; HTCA defect; impaired nuclear shaping
Nup188	Inner ring	#102650 (VDRC)	Normal
**Nup133**	**Y-complex**	**#58290 (Bloomington)**	**Early HTCA defect; impaired nuclear shaping**
		**#110194 (VDRC)**	**Early HTCA defect; impaired nuclear shaping**
Nup37	Y-complex	#62328 (Bloomington)	Normal
Nup75	Y-complex	#28315 (Bloomington)	Normal
Nup160	Y-complex	#32391 (Bloomington)	Normal
Nup44A (Seh1)	Y-complex	#32942 (Bloomington)	Normal
Sec13	Y-complex	#32468 (Bloomington)	Normal
Nup43	Y-complex	#33645 (VDRC)	Meiotic defect; late HTCA defect; impaired nuclear shaping
		#108595 (VDRC)	Severe meiotic defect; HTCA defect

bioRxiv preprint doi: https://doi.org/10.64898/2026.04.28.721503; this version posted May 1, 2026. The copyright holder for this preprint (which was not certified by peer review) is the author/funder. This article is a US Government work. It is not subject to copyright under 17 USC 105 and is also made available for use under a CC0 license.

**Nup107**	**Y-complex**	**#22407 (VDRC)**	**Early HTCA defect; impaired nuclear shaping**
		**#110759 (VDRC)**	**Early HTCA defect; impaired nuclear shaping; minor meiotic defect**
ELYS	Y-complex	N/A	N/A
Mbo (Nup88)	Cytoplasmic filaments	#77374 (Bloomington)	Multiple centrioles/meiotic defect
Nup214	Cytoplasmic filaments	#33897 (Bloomington)	Meiotic defect/multiple centrioles; HTCA defect
Nup358	Cytoplasmic filaments	#34967 (Bloomington)	No spermatocytes
		#38581 (VDRC)	Severe meiotic defect; HTCA defect
		#38583 (VDRC)	Normal
CG18787 (NLP1)	Cytoplasmic filaments	#55375 (Bloomington)	Normal
Rae1	Peripheral	#32882 (Bloomington)	Severe meiotic defect; impaired nuclear shaping; HTCA defect
